# The Role of Cystinosin in the Intermediary Thiol Metabolism and Redox Homeostasis in Kidney Proximal Tubular Cells

**DOI:** 10.3390/antiox7120179

**Published:** 2018-12-03

**Authors:** Rodolfo Sumayao, Philip Newsholme, Tara McMorrow

**Affiliations:** 1 Chemistry Department, De La Salle University, Manila 1004, Philippines; rodolfo.sumayao@dlsu.edu.ph; 2 School of Pharmacy and Biomedical Sciences and Curtin Health Innovation Research Institute (CHIRI), Curtin University, Perth 6845, Australia; 3 Conway Institute, School of Biomolecular and Biomedical Science, University College Dublin, Dublin 4, Ireland

**Keywords:** cystinosin, cystinosis, cysteine, cystine, cysteamine, glutathione, kidney, proximal tubule, lysosome, redox, thiol, oxidative stress

## Abstract

Cystinosin is a lysosomal transmembrane protein which facilitates transport of the disulphide amino acid cystine (CySS) from the lysosomes of the cell. This protein is encoded by the CTNS gene which is defective in the lysosomal storage disorder, cystinosis. Because of the apparent involvement of cystinosin in the intermediary thiol metabolism, its discovery has fuelled investigations into its role in modulating cellular redox homeostasis. The kidney proximal tubular cells (PTCs) have become the focus of various studies on cystinosin since the protein is highly expressed in these cells and kidney proximal tubular transport dysfunction is the foremost clinical manifestation of cystinosis. The lysosomal CySS pool is a major source of cytosolic cysteine (Cys), the limiting amino acid for the synthesis of an important antioxidant glutathione (GSH) via the γ-glutamyl cycle. Therefore, loss of cystinosin function is presumed to lead to cytosolic deficit of Cys which may impair GSH synthesis. However, studies using in vitro models lacking cystinosin yielded inconsistent results and failed to establish the mechanistic role of cystinosin in modulating GSH synthesis and redox homeostasis. Because of the complexity of the metabolic micro- and macro-environment in vivo, using in vitro models alone may not be able to capture the complete sequence of biochemical and physiological events that occur as a consequence of loss of cystinosin function. The coexistence of pathways for the overall handling and disposition of GSH, the modulation of CTNS gene by intracellular redox status and the existence of a non-canonical isoform of cystinosin may constitute possible rescue mechanisms in vivo to remediate redox perturbations in renal PTCs. Importantly, the mitochondria seem to play a critical role in orchestrating redox imbalances initiated by cystinosin dysfunction. Non-invasive techniques such as in vivo magnetic resonance imaging with the aid of systems biology approaches may provide invaluable mechanistic insights into the role of cystinosin in the essential intermediary thiol metabolism and in the overall regulation cellular redox homeostasis.

## 1. Introduction

Cystinosin is a lysosomal transmembrane protein whose chief function is to transport CySS from the lysosomal compartment to the cytosol [1,2,3,4,5,6]. CySS is a disulfide amino acid that is generated from the oxidation of the sulfhydryl groups of two Cys molecules via the formation of disulphide bonds. The discovery of cystinosin and its function in 2001 [4,7] has attracted many researchers to further investigate its regulation, interaction with other proteins and its role in cellular homeostasis, especially in kidney PTCs. Cystinosin is more widely known for its key involvement in the molecular pathogenesis of the lysosomal storage disorder, cystinosis (MIM 21,980). Cystinosis is a rare autosomal recessive disorder characterized by the excessive accumulation of a CySS in the lysosomes of the cell [1,2,8]. The estimated incidence of cystinosis is 1 in 100,000 to 200,000 live births and has been documented worldwide [1,2]. The disease is caused by mutations in the CTNS gene which encodes for cystinosin [1,2,3,8]. The discovery of the CTNS gene has provided the fundamental basis for various molecular groundwork in cystinosis. These include the identification of different mutations in CTNS gene associated with different clinical variants of cystinosis and the molecular characterization of cystinosin.

The multi-systemic effects of the loss of cystinosin function or its deficiency in cystinosis patients fuelled investigations into the role of cystinosin in cellular homeostasis, most especially in the kidneys being the most vulnerable organ in the natural evolution of the disease [1,2,9,10]. Kidney dysfunction associated with cystinosis clinically manifests as excessive wasting of water, electrolytes, minerals and essential nutrients into the urine, a clinical condition known as renal Fanconi syndrome [1,2,8,9]. Infants with the classic nephropathic cystinosis show signs of renal Fanconi syndrome as early as six months of age which leads to failure to thrive in affected children [1,2]. Despite the plethora of studies on the molecular characteristics and function of cystinosin, its role in maintaining cellular integrity and tissue function remains to be established. This review article examines the current status of our understanding on the role of cystinosin in the cellular intermediary thiol metabolism. Towards this goal, the potential role of cystinosin in modulating cellular redox homeostasis (or vise versa) and its impact on cellular integrity and tissue function are discussed, with special emphasis on kidney proximal tubules.

## 2. Historical Aspects

The probable existence of a lysosomal CySS transporter was first recognized in the late 1960s through the differential centrifugation studies performed on cystinotic leukocytes [11,12] and fibroblasts [13] which demonstrated that large amounts of CySS co-sedimented with lysosomal enzymes. Since no CySS-reducing systems were known to exist in the lysosomes at that time, it was hypothesized that defective CySS transport may have been the cause of CySS accumulation in cystinosis patients. This hypothesis was reinforced in the 1980’s through the studies performed on whole lysosomes artificially loaded with high concentrations of CySS using a lysomotropic agent CySS dimethyl ester. Various studies independently demonstrated rapid CySS egress from normal lysosomes, whereas this activity was abolished in cystinotic lysosomes [14,15,16]. Furthermore, the latter studies have provided clues into the existence of an unknown CySS transporter because CySS efflux exhibited saturation kinetics [16] and CySS counter transport was demonstrated across the lysosomal membrane [17]. It thus became apparent that the excessive accumulation of CySS in the lysosomes, associated with cystinosis, was due to a defective lysosomal transporter that facilitates CySS exodus from the lysosomal compartment. 

## 3. Molecular Characterization of Cystinosin

**Figure 1:**
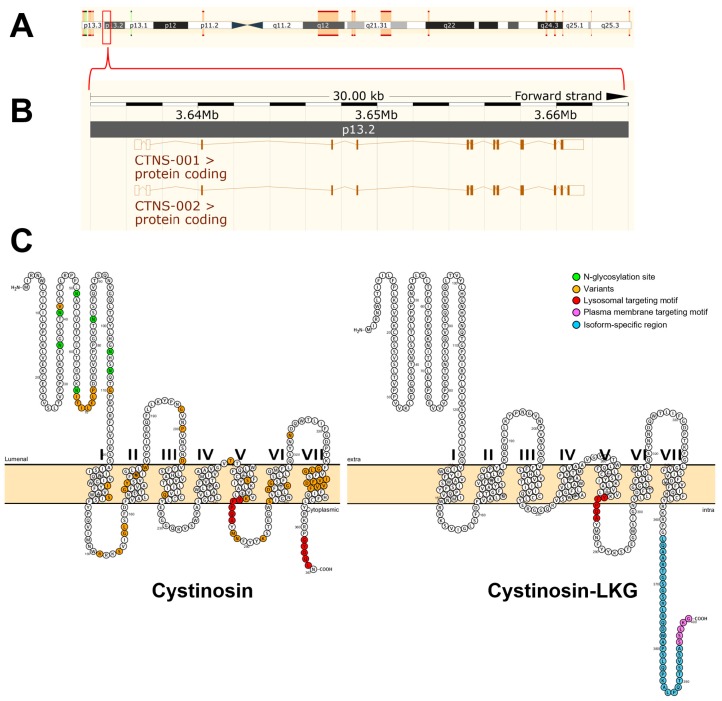
The CTNS gene and the predicted molecular structure of cystinosin. The CTNS gene was mapped to the short arm of chromosome 17 (17p13) using linkage analysis (**A**). The gene contains 12 exons distributed across approximately 23 kb of genomic DNA (**B**). The first two of which are noncoding. The remaining 10 exons encode the 367 amino acids that make up cystinosin. Using the open-source tool for visualization of proteoforms, PROTTER [20], the two cystinosin isoforms were visualized (**C**). The canonical cystinosin isoform (UniProt #O60931-1) is predicted to contain seven transmembrane domains, a 128 amino acid N-terminal region bearing seven potential *N*-glycosylation sites (green-colored circles), a non-cleavable signal peptide, and a 10 amino acid cytosolic C-terminal tail. Targeting of cystinosin to the lysosomes requires a signal peptide. At least two lysosome-targeting motifs have been identified. The cytosolic C-terminal domain of cystinosin resides a tyrosine-based GYDQL motif (red-colored circles, residue 362–366). The second motif, termed YFPQA (red-colored circles, residues 281–285), was mapped to the third predicted cytoplasmic loop. Some of the identified mutations are shown (yellow circles). These mutations include missense mutations, in-frame deletions, and insertions which account for different clinical variants of cystinosis. An alternative splicing of the exon 12 that removes the GYDQL motif generates the second isoform of cystinosin (UniProt #O60931-2). This second cystinosin isoform is termed cystinosin-LKG based on its last three amino acids. Cystinosin-LKG differs from the canonical isoform in the carboxy-terminal sequence (cyan-colored circles) and its proposed motif critical for the protein sorting to the plasma membrane, SSLKG (purple-colored circles). Both (**A**,**B**) were generated using the open-source visualization tool, Ensembl 2018 [21].

The Mendelian pattern of inheritance was already recognized in the first cases of cystinosis that were reported but the causative gene was not identified until 1998 by positional cloning [7]. The gene responsible for cystinosis, known as CTNS, was mapped to the short arm of chromosome 17 (17p13) using linkage analysis [18]. To determine the precise genomic organization of CTNS, the region of human chromosome 17p13 encompassing the CTNS gene was mapped and sequenced [19]. The gene contains 12 exons distributed across approximately 23 kb of genomic DNA, first two of which are noncoding [7] (Figure 1). The remaining 10 exons encode the 367 amino acids that make up cystinosin (Figure 1). 

To date, more than 90 mutations have been described in all forms of cystinosis [3]. Some of the mutations in the CTNS gene are shown in Figure 1 which account for different clinical variants and severity of cystinosis. These include missense mutations, in-frame deletions and insertions. There are no mutational hotspots but, by far, the most prevalent is the 57-kb deletion [3,6,7,19]. This mutation accounts for approximately of 50% of individuals with nephropathic cystinosis in the United States and Northern European populations [1,2]. This founder mutation encompasses the first nine exons and introns of CTNS gene and interrupts exon 10 leading to the complete loss ofof cystinosin expression [1,2,3,6,19]. Within the group of individuals with nephropathic cystinosis, truncating CTNS mutations, as well as the 57-kb deletion, result in a severe, infantile form of the disease [1,2]. In individuals with intermediate and non-nephropathic cystinosis, the 57-kb deletion may be present in heterozygous state, along with a more benign mutation [1,22,23,24]. Presumably, these mutations allow some residual CySS transport, accounting for mild clinical presentation of the disease [1,6]. 

The crystal structure of cystinosin has not been generated to date. Human cystinosin is predicted to contain seven putative transmembrane domains, a large 128-amino acid N-terminal domain, which is oriented towards the lysosomal lumen, and a short 10-amino acid C-terminal tail, which resides in the cytosol (Figure 1). The N-terminal domain possesses seven potential *N*-glycosylation sites and a non-cleavable signal peptide, while the C-terminal domain contains a tyrosine-based sorting motif required for its delivery to lysosomes [1,7,19]. 

Transfection and co-localization studies of a cystinosin-enhanced green fluorescent protein (pCTNS-EFGP) fusion plasmid construct revealed that cystinosin is localized to the lysosomes [4]. Furthermore, immunohistochemical studies using anticystinosin antibodies demonstrated abundance of cystinosin in kidney PTCs, which appeared microgranular and diffusely distributed within the cytoplasm [25]. In contrast, lower expression of cystinosin was only observed in distal tubules and glomeruli. This differential localization pattern suggests that cystinosin is highly required in PTCs but not in glomeruli and may explain why proximal tubulopathy generally manifests at a very early stage of cystinosis, whereas glomerular dysfunction appears only later in the course of the disease [3].

Targeting of cystinosin to the lysosomes requires a signal peptide. At least two lysosome-targeting motifs have been identified (Figure 1). The cytosolic C-terminal domain of cystinosin contains a tyrosine-based GYDQL motif (residue 362–366) [4], which resembles the classic tyrosine-based lysosomal targeting motif GY-XX-Φ (X is any amino acid; Φ is a hydrophobic amino acid) [26]. Mutation or deletion of the GYDQL motif, in particular of tyrosine (Y) or leucine (L) residues, results only in the partial redirection of cystinosin to the plasma membrane. The second motif YFPQA (residue 281–285) was mapped to the third predicted cytoplasmic loop by site-directed mutagenesis [4]. Point mutations in the YFPQA motif did not affect the localization of cystinosin, suggesting that the YFPQA sequence may not be obligatory for the targeting of cystinosin to the lysosomes [6]. It appears that the GYDQL and YFPQA motifs follow a co-dependency relationship since deletion of the YFPQA pentapeptide, coupled to the deletion of the GYDQL motif, resulted in a complete relocalization of cystinosin to the plasma membrane [4]. Although the YFPQA motif appears to be not critical in the lysosomal targeting of cystinosin, it was suggested that this motif forms a novel conformational motif in the fifth inter-transmembrane loop, likely as a part of its secondary structure [6]. 

Another cystinosin isoform was identified and termed cystinosin-LKG based on its last three amino acids (Figure 1). This isoform is generated by an alternative splicing of exon 12 that removes the GYDQL motif [27]. Unlike the canonical isoform, transient transfection experiments of cystinosin-LKG-GFP fusion plasmids in a kidney PTC line, HK-2, revealed that cystinosin-LKG is not only restricted to the lysosomes but is also expressed in the plasma membrane and in other cytosolic structures [27]. Site-directed mutagenesis experiments showed that the carboxyl-terminal SSLKG sequence of cystinosin-LKG is essential for the efficient targeting of this protein to the plasma membrane where it mediates H^+^-coupled CySS transport [28]. Cystinosin-LKG expression is ubiquitous and represents 5–20% of CTNS transcripts, with the exception of the testis in which both isoforms are expressed in equal proportions [29]. Cystinosin-LKG was found to be highly expressed in renal PTCs, pancreatic islets of Langerhans, Leydig cells of the testis, mucoserous glands of the bronchial wall, melanocytes and keratinocytes [29]. The high expression of cystinosin-LKG in these cells and tissues appears to coincide with the clinical features of cystinosis such as renal Fanconi syndrome, male infertility, diabetes mellitus, and hypopigmentation [29].

## 4. Function of Cystinosin

The molecular function of cystinosin was determined by deleting the C-terminal GYDQL sorting motif (cystinosin-ΔGYDQL), therefore redirecting cystinosin to the plasma membrane of COS cells [5]. Using this strategy, the ability of cystinosin to transport CySS could be examined in whole cells resembling a giant ‘inside-out’ lysosomes [5]. These cells expressing cystinosin-ΔGYDQL was demonstrated to selectively take up L-CySS from the extracellular medium at acidic pH. However, CySS transport activity was abolished when the transmembrane pH gradient is disrupted between the acidic extracellular medium and neutral cytosol. These observations indicate that cystinosin co-transports CySS and H^+^, hence, cystinosin operates as a CySS-H^+^ symporter [5]. Thus, this cellular model strongly supports previous studies on lysosomal CySS transport in which the lysosomal H^+^-translocating ATPase promotes the acidification of lysosomal lumen which actively drives the cystinosin-mediated CySS transport in the efflux direction [14,30,31]. Furthermore cystinosin-ΔGYDQL were shown to be highly specific for L-CySS and do not transport other amino acids, including the Cys, which distinguishes it from known plasma membrane CySS transporters, 4F2hc/xCT [32] and b^0,+^AT-rBAT [33]. The absence of homology to any known transporters and the predicted seven transmembrane domain topology suggest that cystinosin defines a novel family of transporters. 

## 5. Cystine Metabolism in the Kidney Proximal Tubules

**Figure 2:**
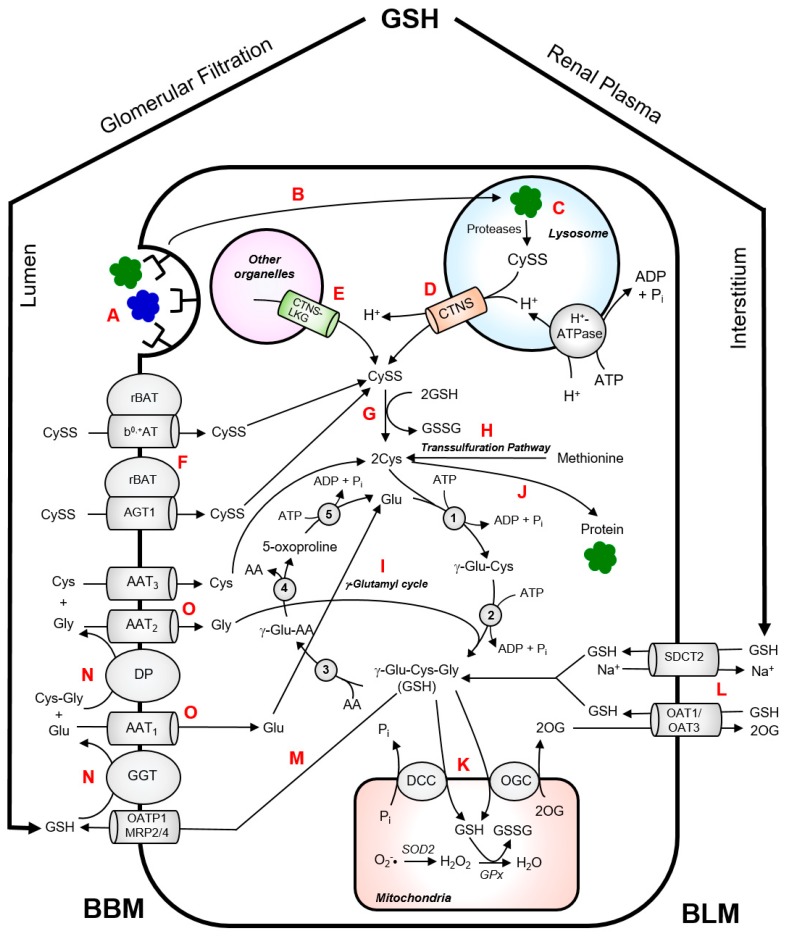
Intermediary thiol metabolism in renal proximal tubular cells. CySS-containing proteins are reabsorbed from the brushborder membrane (BBM) of the PTCs via receptor-mediated endocytosis (A). The CySS-containing proteins enter the lysosomal compartment via fusion of protein-loaded transport vesicles with the lysosomes (B). These proteins undergo cathepsin-catalyzed degradation which generates CySS (C). Cystinosin facilitates the proton-driven efflux of CySS from the lysosomes into the cytosol (D). Cystinosin-LKG can also facilitate exodus of CySS from other cytosolic compartments (E). Non-protein CySS can be taken up by PTCs through heterodimeric CySS transporters, b^0,+^AT-rBAT and AGT1rBAT, and contributes to the cytosolic CySS pool (F). In the cytosol, CySS is rapidly reduced to Cys by cytosolic reducing systems (G). Alternatively, Cys can also be produced via trans-sulfuration pathway (H). The Cys generated in the cytosol can be used for the synthesis of GSH via γ-glutamyl cycle (I) or synthesis intracellular proteins (J). Most of the GSH in the mitochondrial matrix is obtained from the cytosolic GSH pool via oxoglutarate carrier (OGC) or dicarboxylate carrier (DCC) (K). In the mitochondria, superoxide radicals (O_2_^−^•) generated from oxidative phosphorylation are converted to hydrogen peroxide (H_2_O_2_) by superoxide dismutase 2 (SOD2). H_2_O_2_ is eventually neutralized to water (H_2_O) by glutathione peroxidase (GPx) which requires GSH as a cofactor. The kidney extracts approximately 80% of GSH from the plasma. Approximately 50% of this GSH enters the PTCs via sodium–dicarboxylate cotransporter 2 (SDCT2) and organic anion transporters 1 and 3 (OAT1/3) at the BLM (L). Glomerular filtration accounts for approximately 30% of the GSH extraction via BBM route. GSH can also undergo turnover by efflux into the lumen via either organic anion transporting polypeptide 1 (OATP1) or multidrug resistance proteins 2 and 4 (MRP2/4) (M). The high activity of γ-glutamyltransferase (GGT) and dipeptidase (DP) in the BBM of PTC facilitates degradation of GSH to its constituent amino acids (N). The constituent amino acids are then taken up by their respective transporters (AAT) (O) and are used for the cytosolic re-synthesis of GSH. Enzymes of the γ-glutamyl cycle: γ-glutamylcysteine synthetase (1); GSH synthase (2); γ-glutamyl transpeptidase (3); γ-glutamyl cyclotransferase (4); 5-oxoprolinase (5).

CySS is normally generated inside the lysosomes via endoproteolysis of disulphide-containing proteins [34]. Wilmer et al. provided an excellent illustration of CySS metabolism in kidney PTCs [35]. In the kidney, almost all of the protein reabsorption takes place in the kidney proximal tubule via receptor-mediated endocytosis from the brushborder membrane of the kidney PTCs (Figure 2). Fusion of protein-containing transport vesicles with the lysososomes allows internalization of the CySS-containing proteins by the lysosomes. These proteins undergo cathepsin-catalyzed degradation which generates CySS in the lysosomes. Cystinosin facilitates the proton-driven efflux of CySS into the cytosol. CySS is rapidly reduced to Cys by cytosolic reducing systems with the concomitant oxidation of GSH to glutathione disulphide (GSSG). The Cys generated in the cytosol can be used for the synthesis of GSH or intracellular proteins. Experimental data on cystinotic leukocytes and fibroblasts showed that part of the lysosomal CySS pool originates from the uptake of extracellular non-protein CySS [12,36]. In kidney PTCs, the uptake of non-protein CySS from the extracellular lumen is primarily mediated by a heterodimeric exchanger, b^0,+^AT-rBAT, encoded by the *SLC7A9* and *SLC3A1* genes, respectively [33,37] (Figure 2). Mutations in these genes cause cystinuria, which is characterized by excessive wasting of CySS and dibasic amino acids into the urine [33,37]. Proteolysis of CySS-containing proteins within the lysosomes contributes largely to the lysosomal CySS pool. An interesting example is bovine serum albumin (containing 17 mol CySS/mol protein), which has been reported to stimulate CySS accumulation in cystinotic fibroblasts when present in culture medium [34].

Although identification and characterization of amino acid transporters in renal PTCs appear to be almost settled, transporters involved in CySS reabsorption remain to be completely elucidated. Most recently, a novel CySS transporter in renal proximal tubule was identified as the ‘missing partner’ of cystinuria-associated apical b^0,+^AT-rBAT [38]. This transporter AGT1, encoded by the *SLC7A13* gene, has long been postulated as the second CySS transporter in the S3 segment of proximal tubules and a possible candidate involved in isolated cystinuria [38]. The AGT1-rBAT heterodimer was shown to transport CySS and as well as aspartate and glutamate [38].

The discovery of cystinosin-LKG adds to the complexity of the intermediary thiol metabolism in renal PTCs. This cystinosin isoform maintains the H^+^-mediated CySS transport activity of the canonical isoform [28]. Cystinosin-LKG localizes to other cytosolic organelles and is expressed in the plasma membrane [28]. Moreover, cystinosin-LKG widespread distribution across various cellular structures suggests that this isoform can participate in the regulation of intermediary thiol metabolism. Of particular note was the observation that CySS depletion can promote upregulation of cystinosin-LKG in the plasma membrane [28]. Therefore, it can be hypothesized that cystinosin-LKG could facilitate CySS uptake from the extracellular medium during conditions when the cystolic Cys becomes depleted. Despite these important discoveries of various transport systems that facilitate CySS reabsorption in renal proximal tubule, the actual physiological contribution of each of this transport system to the thiol availability and their influence on intermediary thiol metabolism remain to be elucidated. 

## 6. Cystinosin Is Modulated by Intracellular Thiol Availability and Redox Status

Previous seminal work on purified lysosomes from cystinotic cells [16,17] and, more recently, the direct demonstration of the ability of cystinosin to transport CySS in the presence of a proton gradient [5] have restricted the function of cystinosin to facilitating CySS efflux from the lysosomes. Studies conducted by Bellomo et al. [39] demonstrated that cystinosin expression is modulated by intracellular thiol availability and redox status. Under thiol-free conditions, HK-2 cells had reduced intracellular Cys and GSH levels, accompanied by an increase in reactive (ROS) production [39]. These alterations correlated with a significant oxidation of Cys/CySS and GSH/GSSG couples with progressive increase in CTNS mRNA levels [39]. The authors also observed a strong correlation between intracellular Cys and CTNS gene expression (*r*^2^ = 0.85, *p* < 0.001) [39]. Moreover, inhibition of GSH synthesis and induction of oxidative stress using L-buthionine sulfoximine and tertbutylhydroperoxide, respectively, both stimulated CTNS mRNA expression in HK-2 cells [39]. Conversely, the effects of thiol depletion on CTNS mRNA levels could be reverted by Cys and GSH precursors, *N*-acetyl-L-cysteine (NAC) and glutathione ethyl ester, respectively [39]. 

In another study, chronic CySS deprivation of HK-2 cells resulted in an increase in the CTNS promoter activity and CTNS mRNA levels with significant stabilization of CTNS mRNA [40]. These were associated with alterations in the intracellular redox potential of Cys/CySS and GSH/GSSG couples toward a more oxidized state. Consistent with Bellomo et al. study [39], treatment of HK-2 cells with NAC successfully reversed all these effects. 

The data above suggest that intracellular thiol availability and redox status may constitute primary stimuli for the activation of cystinosin expression. That is, the cell may sense changes in its intracellular thiol levels or redox state which, in turn, may respond to these changes by modulating cystinosin expression [39,40]. Cystinosin activation may also be crucial to mobilize lysosomal CySS stores under stressful conditions to provide sufficient Cys in the cytosol in an attempt to maintain a normal cellular redox state. The mechanisms behind the thiol- and redox-mediated modulation of cystinosin expression are not completely understood. It appears that it involves enhancement of CTNS promoter activity with subsequent stabilization of CTNS mRNA [40]. However, because of the complex regulatory elements and molecular crosstalks in vivo, it is very likely that it involves coordinated action of a set of genes that encode for transcription factors, enzymes, or cell transporters that are sensitive to cell stress induced by amino acid depletion or redox perturbations [40].

The identification of the cystinosin-LKG isoforms again adds further complexity to the regulation CySS efflux from various intracellular organelles. As stated previously, cytosolic CySS depletion can promote upregulation of cystinosin-LKG in the plasma membrane [28]. Therefore, this non-canonical form of cystinosin may also actively participate in sensing thiol/disulfide redox status. However, the actual physiological contribution of this cystinosin isoform in maintaining a healthy cellular redox state remains to be established. Recently, it was demonstrated that cystinosin-LKG appears to have similar functional activity as the canonical cystinosin isoform and is essential in the viability of renal PTCs [28].

## 7. Role of Cystinosin in Cellular Glutathione Homeostasis

GSH is a tripeptide consisting of the amino acids glutamate (Glu), Cys and glycine (Gly). Unlike most proteins and peptides, the Glu and Cys residues of GSH are linked through a non-canonical amide bonding between the γ-carboxyl group of Glu and the α-amino group of Cys. This isopeptide bond is resistant to proteases making GSH practically ubiquitous in biological systems [41]. GSH is the most abundant intracellular redox buffer and the main antioxidant that protects cells against oxidative stress [35,42,43,44]. GSH plays numerous roles such as a substrate or cofactor for ROS and drug metabolizing enzymes [41,45]. For example, GSH serves as an important cofactor for the GSH peroxidase family of enzymes which metabolize hydrogen peroxide and lipid peroxides, preventing oxidative damage to cellular components. GSH also plays an important role in regulation of cell growth, proliferation and stress responses [41]. 

Cys is the rate-limiting amino acid in *de novo* synthesis of GSH [35,41]. A significant part of the intracellular Cys pool is utilized for the synthesis of GSH through the γ-glutamyl cycle (Figure 2). This process takes place in the cytoplasm and is catalyzed by two sequential ATP-dependent enzymes, namely, γ-glutamylcysteine synthetase and GSH synthetase [41,45]. While most cells can synthesize GSH, the rates of GSH production may not be sufficient to maintain normal cellular concentrations of GSH during certain disease or stressful state. The observation of elevated urinary levels 5-oxoproline, an intermediate of γ-glutamyl cycle, in untreated cystinosis patients [46] have prompted cystinosis researchers to focus on perturbations in GSH synthesis in an attempt to explain the pathophysiology of cystinosis. 

Assuming that the CySS originating from the lysosomes supplies the majority of Cys in the cytosol which, in turn, is used for the synthesis of GSH, a deficiency or absence of cystinosin may, in theory, result in cytosolic deficit in GSH. However, the measurement of GSH levels in various in vitro models of cystinosis has yielded inconsistent results. Earlier studies on the role of cystinosin on GSH metabolism were conducted in various cell types obtained from cystinosis patients. Reduced total GSH content was observed in cystinotic skin fibroblasts [47] and kidney PTCs [48]. In contrast, Manucci et al. [49] reported normal basal GSH levels in human cystinotic fibroblasts but these cells exhibited a more pronounced increase in pyroglutamate levels while GSH decreased to lower levels compared to normal cells. In addition, human cystinotic fibroblasts had decreased capacity for GSH synthesis following exposure to oxidative stress and upon inhibition of ATP synthesis [49]. These results suggest that the activity of ATP-dependent γ-glutamyl cycle may be compromised under conditions of cystinosin deficiency. On the other hand, we and others reported that transient silencing of CTNS gene in kidney PTC line, HK-2, resulted in a dramatic reduction of total intracellular GSH and altered GSH/GSSG redox potential towards a more oxidized state [39,50]. Interestingly, in one study, although no reduction in GSH was observed in cultured cystinotic fibroblasts, GSH levels were reported to be inversely correlated with total intracellular CySS levels [51], suggesting that CySS in the lysosomes may possibly be a limiting factor for the synthesis of GSH in the cystosol. Conversely, examination of the fetal and non-fetal skin and lung-derived fibroblasts obtained from cystinosis patients revealed normal [GSH]/[GSSG] ratio under steady-state conditions and following inhibition of trans-sulfuration pathway and CySS/Glu antiporter system xc^−^ [52], suggesting that the loss of cystinosin function does not lead to cytosolic redox perturbations, at least in cystinotic cells in culture. GSH levels were found to be normal in cystinotic HPV 16 E6/E7-immortalized PTC line [53]. Similarly, total GSH content in simian virus 40 T antigen/human telomerase reverse transcriptase (SV40 T/hTERT) conditionally immortalized renal PTCs obtained from the urine of cystinosis patients was comparable with healthy controls [54]. Interestingly, GSSG were significantly increased in these cells and correlated with intracellular CySS levels, which resulted in the alteration of GSH redox status. In several other studies, no substantial alterations were observed in either GSH levels or γ-glutamyl cycle enzyme activities in cystinotic fibroblasts [55,56,57,58]. These contradicting observations suggest that alternative mechanisms exist to circumvent cystinosin dysfunction in an attempt to maintain cellular redox homeostasis. However, the metabolic interconnections in vivo may add a daunting complexity to a scenario where cells try to cope with disruption ofredox homeostasis as a consequence of loss of cystinosin function. 

Inconsistencies in the observations using in vitro models may be attributed to metabolic differences between various cell types studied and differences in experimental conditions [35]. For example, differences in cell culture conditions pertaining to the composition of culture medium (i.e., levels of thiols and serum) may cause discrepancies in thiol metabolism, hence, may affect the intracellular GSH levels. However, in majority of the in vitro studies cited, the levels of thiols in cell culture medium were not indicated. In one study, however, CTNS mRNA levels and GSH/GSSG and Cys/CySS redox potentials were significantly altered in HK-2 cells in the absence thiols in cell culture medium [39], which indicates that intracellular thiol availability can modulate CTNS gene expression and redox status. 

**Figure 3:**
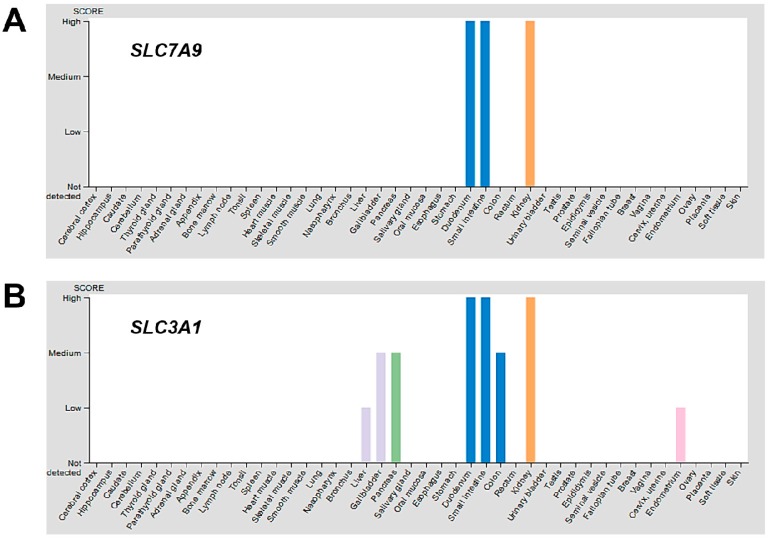
Protein expression overview of known CySS transporters in various tissues. Using the Human Protein Atlas [59], the protein expression overview of CySS transporters b^0,+^AT and rBAT, encoded by the *SLC7A9* (**A**) and *SLC3A1* (**B**) genes, respectively, were visualized. The kidneys (orange bar) represent one of the tissues with the highest expression of *SLC7A9* and *SLC3A1*. The skin, liver, lung, muscles, thyroid and other tissues minimally or do not express the same transporters. Only CySS transporter genes with available protein data for which a knowledge-based annotation gave conclusive results were included.

Different cells and tissues may also respond differently to changes in intracellular thiol or redox status by modulating a set of genes that encode for amino acid transporters to compensate for the loss of cystinosin. The protein expression overview of the Human Protein Atlas [59] indicates the kidneys represent one of the tissues with the highest expression of disulphide-linked CySS transporters, b^0,+^AT and rBAT, encoded by the *SLC7A9* and *SLC3A1* genes, respectively, while skin, liver, lung, muscles, thyroid and other tissues minimally or do not express the same transporters (Figure 3). It is possible that the levels of expression of different Cys/CySS transporters in various cell types and tissues may produce differential responses to intracellular thiol depletion and/or redox perturbations. 

Differences in the techniques employed in the measurement of GSH concentration may also impact estimation of GSH content. Majority of the in vitro studies cited employed high performance liquid chromatography (HPLC) for the measurement of GSH in cell lysates. HPLC is regarded as the ‘gold standard’ for the measurement of GSH and GSSG in biological samples and is more sensitive than colorimetric techniques. A HPLC method coupled with UV detection employed for the measurement of GSH species in A549 cells yielded a detection limit of 1 µM [60]. On the other hand, measurement of GSH species using an enzyme recycling method coupled with microplate-based UV detection had a detection limit of 103 µM [44]. The differences in sensitivity and specificity of these techniques may present discrepancies in the estimation of GSH concentration in biological samples.

## 8. Alternative Rescue Mechanisms to Remediate Absence of Cystinosin 

The use of in vitro models may underestimate the high metabolic activity of kidney PTCs in vivo which almost exclusively depends from mitochondrial oxidative phosphorylation for ATP production [35]. This results in a higher ROS production in vivo and may render cystinosin-deficient cells more vulnerable to oxidative stress. Furthermore, because of the complex metabolic macro- and micro-environment in vivo, it is also likely that other mechanisms may be involved to rescue the cells from GSH deficiency as a consequence of reduction or absence of cystinosin function. 

The kidneys highly depend on adequate supply of GSH to maintain a normal GSH/GSSG ratio which is critical to protection against damaging ROS and to maintenance of normal function. Central to the unique physiology of the kidney, in particular the proximal tubule, with respect to GSH homeostasis lies in the coexistence of pathways for synthesis, degradation, efflux and uptake of GSH [45]. Intracellular GSH synthesis alone may not be sufficient to maintain intracellular GSH concentrations especially during pathological states, such as cystinosis. Aside from the intracellular synthesis, renal PTCs can obtain GSH from the extracellular space, via transport across the basolateral membrane (BLM) (Figure 2). The organic ion transporters OA1 and OAT3 and the sodium–dicarboxylate 2 exchanger SDC2 represent major transport systems for the uptake of GSH from BLM of renal PTCs which help in maintaining intracellular GSH levels [41,45]. 

As with many tightly regulated compounds, GSH degradation in the kidney occurs separately from its synthesis and occurs primarily in the proximal tubule. The enzymes required for GSH degradation are found extracellularly, specifically in the luminal or brush border membrane (BBM) of renal PTCs. As shown in Figure 2, a part of the intracellular GSH undergoes turnover by being transported into the lumen via organic anion transporting polypeptide 1 (OATP1) or multidrug resistance proteins 2 and 4 (MRP2/4). The BBM of renal PTCs possesses an ectoenzyme γ-glutamyltransferase (GGT) which catalyzes the initial cleavage of the γ-glutamyl peptide bond between the Gluand Cysresidues of GSH. This is followed by the hydrolysis of peptide bond between the Cysand Glyresidues, which is catalyzed by the enzyme dipeptidase (DP). The constituent amino acids are then reabsorbed into the renal PTCs by various amino acid transporters. Cys can then be used for the synthesis of intracellular proteins or for the intracellular re-synthesis of GSH. In theory, the high GGT activity in renal PTCs will practically degrade all luminal GSH and should supply the PTCs with sufficient Cys for intracellular GSH synthesis [45]. Therefore, under pathological conditions such as cystinosin dysfunction, PTCs may resort to redirecting GSH towards the renal circulation to effectively enable its turnover.

The high energy demands and chronic exposure of the kidney to ROS necessitate a continuous supply of antioxidants to maintain its normal function. Indeed, the kidneys extract approximately 80% of the plasma GSH pool during a single pass of the blood through the renal circulation [45]. Approximately 50% of GSH removal from renal plasma occurs via BLM route with the aid of GSH transporters, OAT1/3 and SDC2 (Figure 2) [45]. Glomerular filtration accounts for approximately 30% of GSH removal via the BBM route through the action of GGT and DP [45]. Moreover, the inter-organ translocation and turnover process of GSH is seen as strategic as this allows transport of Cys in its stable form and its regeneration inside the cell facilitates re-synthesis of GSH. These mechanisms occurring in proximity with each other is critical as Cys constitutes the limiting precursor for the *de novo* synthesis of GSH. Cys is also relatively unstable in aqueous solutions at physiological pH, undergoing relatively facile autooxidation to CySS, which is relatively insoluble. 

These rescue mechanisms may compensate for the lack of cystinosin by orchestrating GSH uptake, efflux and degradation to maintain cellular redox homeostasis. However, under pathological conditions, it is also possible that the rate of GSH turnover in vivo may not be adequate or sufficient to maintain redox homeostasis and sustain high energy demands of renal PTCs where solute and nutrient reabsorption depend on cell energy and redox state. This may depend on the nature of redox imbalance and other key players in vivo that may be involved in the cascade of events initiated by loss of cystinosin function. Therefore, to shed light on the complexity of the metabolic interconnections in vivo, it may be useful to conduct genetic manipulation studies such as knockout of specific amino acid transporters and other genes involved in the thiol metabolic network to determine their precise contribution and mechanistic involvement in the modulation of intermediary thiol metabolism and cellular redox homeostasis in health and pathological conditions. Indeed, it was previously demonstrated that CySS deprivation induces the transcription of the CySS/Glu transporter through a specific amino acid responsive element (AARE) sequence located in its promoter [61]. Interestingly, the same AARE exists in the second intron of the CTNS gene and may play a crucial role in the regulation of the CTNS gene [40].

## 9. The Role of Cysteamine

Cysteamine is a small aminothiol endogenously derived from the degradation of coenzyme A via a highly conserved pathway [62]. In mammals, the membrane-associated enzyme pantetheinase catalyzes the hydrolysis of the pantetheine moiety of coenzyme A to produce cysteamine and pantothenic acid [62]. For decades, cysteamine, in the form of cysteamine bitartrate, has been employed for the treatment of cystinosis. The CySS depletion therapy with cysteamine has revolutionized the management and prognosis of nephropathic cystinosis and has greatly improved the quality of life of cystinosis patients [63]. Cysteamine therapy has been shown to retard renal and non-renal complications associated with cystinosis and has improved linear growth in cystinotic children [1,2].

Cysteamine enters the lysosomes through a distinct transporter which is still unknown to date [63]. In the lysosomes, cysteamine breaks down CySS and combines with Cys to form a mixed disulfide cysteamine-Cys which exits the lysosomes through a cationic amino-acid exporter PQLC2 [1,63]. The cysteamine-Cys disulfide is then reduced to cysteamine and free Cys by cytosolic reducing systems. This process permits the cycling of cysteamine between the lysosomes and cytoplasm, with each cycle removing 1 mole of half CySS per mole of cysteamine [1].

The mechanisms of protection by cysteamine remains unclear. In our previous study, treatment with cysteamine dramatically increased the intracellular GSH levels and reduced the oxidative stress index in HK-2 cells following small interfering RNA (siRNA)-mediated CTNS gene inhibition [50]. Consistent with our findings, cysteamine also increased the GSH content in conditionally immortalized PTCs exfoliated from the urine of cystinosis patients [54]. It appears that aside from the CySS depletion afforded by cysteamine, it can augment intracellular thiol availability which can promote GSH synthesis and enhance the cells’ antioxidant capacity. Indeed, a related aminothiol, NAC, can alter the GSH/GSSG and Cys/CySS couple toward a more reduced state in cultured PTCs [39], presumably by increasing Cys availability which enhances GSH synthesis. 

Although cysteamine remains the institution of therapy for cystinosis and has revolutionized the clinical management of cystinosis patients, it does not treat the proximal tubulopathy associated with the disease and renal Fanconi syndrome still occurs [64]. This suggests that cystinosin may have other roles critical to the function and integrity of renal PTCs. 

## 10. The Role of Mitochondria in Modulating Redox Homeostasis

The high rates of aerobic metabolism in the kidney, particularly in the proximal tubules, makes it chronically exposed to high levels of ROS which can potentially induce cellular damage, particularly in the mitochondria. The mitochondria generate high levels of ROS during oxidative phosphorylation which necessitates high concentration of GSH in the mitochondrial matrix. This has important implications for cellular energetics and in regulation of cellular and mitochondrial redox status. The mitochondria obtain most, if not all, of its GSH supply from the cytoplasmic GSH pool to maintain normal function. In the renal proximal tubule, the mitochondrial GSH pool represents approximately 15–30% of the total cellular GSH content [45]. GSH transport into the mitochondrial matrix is mediated by the dicarboxylate and 2-oxoglutarate transporters in exchange for oxoglutarate (2-OG) or inorganic phosphate (P_i_) (Figure 2) [41,45]. Mitochondrial GSH transport is discussed in great detail elsewhere [41,45].

Mitochondrial anomalies have long been implicated in the pathogenesis of cystinosis. A striking degree of abnormal mitochondrial morphology, mitochondrial autophagy and augmented oxidative stress were observed in cystinotic kidney PTCs [65]. Most recently, it was demonstrated that *Ctns* −/− mice and the PTCs isolated from them exhibit extensive mitochondrial oxidative stress [66]. The mitochondria-targeted superoxide dismutase mimetic, mito-TEMPO, repaired dysfunctional mitochondria and improved epithelial function and integrity in these mice, presumably by attenuating the overproduction of ROS in the mitochondria. In agreement with these observations, we observed a dramatic reduction in the mitochondrial transmembrane potential and ATP content in *Ctns* −/− murine PTCs [67]. 

The role of mitochondria in apoptosis is well established through the mitochondrial dependent pathways of cell death. These observations underscore the role of mitochondria in orchestrating cellular demise as a consequence of cystinosin dysfunction. We hypothesize that cystinosin deficiency may lead to compensatory increases in mitochondrial GSH transport and content but may be insufficient to counteract the overwhelming oxidative stress induced by cystinosin dysfunction. If this is not rectified, it may lead to a cascade of events that will ultimately result to mitochondrial dysfunction and cell death. Interestingly, it appears that the swan-neck lesions in the renal proximal tubule associated with cystinosis is an adaptation to oxidative stress and these lesions develop concurrently with the loss of mitochondria with increased mitochondrial superoxide production [68]. These aberrations can be delayed by treatment with a mitochondria-targeted antioxidant, MitoQ. This suggests that maintenance of mitochondrial GSH pool is critical for cell survival since solute transport across the apical membrane of renal PTCs depend on the cellular redox and energy state which concurrently allow the cells to reabsorb essential solute and nutrients to generate energy and sustain normal function [35].

## 11. Conclusions and Future Directions

Although in vitro model systems lacking cystinosin function have shed some light on the role of cystinosin in cellular redox homeostasis, the complexity of metabolic interconnections that occur in vivo suggests that the level and activity of cystinosin can be impacted by several intracellular and extracellular stimuli. In particular, with respect to GSH homeostasis, the kidney PTCs possess an intricate mechanism for the overall disposition and handling of GSH primarily because of the coexistence of pathways for synthesis, degradation, efflux and uptake of GSH. Although cystinosin can respond as necessary to alterations in Cys/Cyss and GSH/GSSG redox couples, the complexity of intracellular redox state and the interactions between these two redox couples suggest a more detailed molecular approach is required to understand the mechanistic role of cystinosin in regulating cellular intermediary thiol metabolism and redox homeostasis. Furthermore, the existence of cystinosin-LKG isoform presents a highly intricate CySS transport system that modulates CySS exodus from various intracellular organelles and may be involved in the overall modulation of thiol metabolism and redox status in kidney PTCs. 

The elaborate network of interconnected metabolic pathways and the possible crosstalk between them in vivo suggest that a more robust model, such as *Ctns* −/− mice [69], is crucial to fully understand the mechanistic role of cystinosin in modulating the overall redox status and functional integrity of renal PTCs. Systems biology approaches based on realistic kinetic data [70] and non-invasive magnetic resonance imaging techniques [71,72] have recently been utilized to gain invaluable insights into the dynamics of thiol metabolism in complex biological systems. The same approaches may be used to better understand the role of cystinosin in the intermediary thiol metabolism and in the overall regulation of redox homeostasis in renal PTCs. Emerging evidences indicate that cystinosin is not merely a CySS transporter. Most recently, it was demonstrated that cystinosin facilitates intracellular trafficking and localization of the autophagy receptor, LAMP2A [73] and loss of cystinosin function leads to altered lysosomal dynamics and autophagy and, ultimately, epithelial dysfunction [66]. Therefore, cystinosin plays a crucial role in the ‘housekeeping’ function of autophagy in renal PTCs, which is critical for the timely removal of dysfunctional or damaged cellular organelles and misfolded or aggregated proteins to maintain cellular integrity. Finally, it appears that mitochondria can receive signals from redox perturbations initiated by cystinosin dysfunction and may orchestrate cell fate. Therefore, cystinosin may have the capacity to influence various key players and cellular pathways that maintain cellular integrity, thus, exploration of these pathways and their interconnections would be a promising challenge for future research and would be of great importance to understanding of the full spectrum of cystinosin function.
